# Addressing the Mental Health Needs of Black American Youth and Families: A Case Study from the EMBRace Intervention

**DOI:** 10.3390/ijerph15050898

**Published:** 2018-05-02

**Authors:** Riana E. Anderson, Shawn C. T. Jones, Crystal C. Navarro, Monique C. McKenny, Tulsi J. Mehta, Howard C. Stevenson

**Affiliations:** 1 Children, Youth, and Families Department, University of Southern California, Los Angeles, CA 90089, USA; ccnavarr@usc.edu; 2 Human Development and Quantitative Methods Design, Graduate School of Education, University of Pennsylvania, Philadelphia, PA 19104, USA; shawnjon@upenn.edu (S.C.T.J.); tulsij@gse.upenn.edu (T.J.M.); howards@gse.upenn.edu (H.C.S.); 3 Department of Educational and Psychological Studies, University of Miami, Coral Gables, FL 33124, USA; mcm370@miami.edu

**Keywords:** clinical intervention, racial socialization, coping, Black families, psychosocial outcomes

## Abstract

Black American youth are vulnerable to the consequences of repeated exposure to racial discrimination, particularly through hampered coping abilities and greater internalizing and externalizing problems. One way in which Black American parents have protected their children from these deleterious consequences is through racial socialization, or communication regarding aspects of racialized experiences and contexts. Less is known, however, about the potential therapeutic benefits of racial socialization via clinical intervention. The five-week Engaging, Managing, and Bonding through Race (EMBRace) racial socialization intervention was developed to enhance coping strategies for parents and adolescents and reduce adolescent internalizing and externalizing problems. The purpose of this study is to describe a case study of one family through a mixed methods approach. Variables of interest included racial discrimination, racial socialization, coping, and psychological well-being. Quantitative and qualitative assessments were performed two weeks prior to and one week after the implementation of EMBRace, with qualitative data collected throughout the intervention. Results indicate a developing sense of coping for the adolescent and parent and reduced adolescent psychosocial problems despite increased racialized stress. Results will be used to further investigate the hypotheses proposed in the pilot with a powered sample, and future studies will explore how sociodemographic and biopsychosocial variables relate to policy recommendations, program implementation, and psychosocial outcomes.

## 1. Introduction

Various policies and practices have been developed in the United States to counteract racial discrimination, that is, when persons are mistreated because of their actual or perceived race [1]. Indeed, the Civil Rights Act of 1964 was meant to stop the systemic racism enforced by Jim Crow Laws and was supposed to shatter the pervasive “separate but equal” ideology. However, over 50 years later, racial discrimination continues to be reported by the vast majority of Black Americans in their immediate environments, including classrooms, work spaces, and neighborhoods [1,2]. Developmentally, Black American youth are just as likely to report experiencing racial discrimination as adults, which is associated with subsequent challenges in coping and psychological well-being [3,4]. Although the literature is robust in the identification of the negative influence of discrimination, Black American parents have challenged its deleterious effects through racial socialization (RS). Racial socialization, or the verbal and nonverbal messages individuals communicate regarding race [5], has been shown to interrupt the association between discrimination and youth outcomes. For example, the relationship between RS, racial identity, and cultural familiarization helps Black American youth to be resilient in developing coping, self-concept, and cognitive appraisal skills [6]. Despite some equivocal findings regarding the relationship between RS, racial discrimination, and various psychological, health, academic, and identity outcomes in Black American youth (e.g., [7]), the vast majority of evidence indicates favorable outcomes for Black American youth when parents provide more frequent RS communication [5].

Given that racial discrimination is as frequently reported today as it was in the Civil Rights era [1], with the majority of Black Americans indicating no progress in their civil rights over their lifetime [8], it is imperative for scholars and practitioners to better understand RS as a tool to buttress discriminatory effects on youth. Applied methods such as clinical interventions may provide greater understanding of the mechanism of change associated with RS [9]. Clinical interventions may help to explicate the practical application of RS and the explicit reduction of psychological distress as it relates to discriminatory experiences. In particular, Stevenson (2014) posits that the frequent and efficacious use of RS helps to mitigate the negative outcomes from racial discrimination through the Racial Encounter Coping Appraisal and Socialization Theory (RECAST; [3]), yet few studies have applied the practices of RS within interventions with Black families. Among the exceptions are the Preventing Long-term Anger and Aggression in Youth (PLAAY; [10]) intervention, in which parents and community members are empowered to address aggressive behavior in children through the use of sports. PLAAY combines the roles of therapist and basketball coach, whereby the clinician will make the children aware of their anger and help them cope with it, especially in light of racialized issues. Despite being able to educate Black American boys on how to manage their anger and maintain a separate parent support group, PLAAY does not teach the child or their parents how to resolve externalizing problems together. Another existing intervention incorporating RS for Black American families is the Black Parenting Strengths and Strategies (BPSS; [11]) program, which focuses on teaching parents how to positively and effectively manage the early development of conduct problems in young children. While the BPSS intervention served as a foundation for directly addressing RS practices with Black American parents, the program does not include sessions for the targeted young child.

In considering the need for appropriate interventions for Black American families addressing the common stressor of discrimination, it is important to recall the record of racial misconduct which has historically distanced Black American individuals from seeking health treatment [12,13]. For example, early “scientific” writing made false claims about Black American individuals having lower intelligence, among other forms of medical malpractice (e.g., forced sterilization, etc.; [13]). Moreover, various clinical factors, including intervention composition, clinician preparation, and intervention content, highlight why appropriate programming may be lacking as it pertains to Black American families coping with discrimination [14]. Franklin (1999) indicated a need for clinicians to have knowledge of the Black American experience to motivate participants to disclose incidents of racial discrimination [15]. In addition, Coard et al. (2007) demonstrated that parenting programs for Black American parents can be successful in community settings, yet also indicated that there has been minimal instruction on how to change existing parenting programs [11]. 

Taken together, The Engaging, Managing, and Bonding through Race (EMBRace; [16]) intervention was developed to meet the needs of Black American families experiencing racial stress by testing RS as a mechanism of change through applied methods and improving upon the clinical problems experienced in prior research. The intervention was developed from the RECAST model, which emphasizes the crucial role RS plays in the coping process of Black American youth experiencing discriminatory stress [3]. In particular, RECAST has three discriminatory racial encounter elements at its core: knowledge (awareness), management (appraisal and reappraisal), and coping (engagement and resolution) processes. Racial socialization is the mechanism through which each of these elements is expressed to other members of the family, especially through modeling. EMBRace takes a novel approach in that it provides a space for the parent and adolescent child to individually engage in therapy to address personal concerns and then brings them together to address how the family unit can best navigate the stress of facing racial discrimination through the RECAST elements. A recent pilot of EMBRace was conducted in the United States with ten Black American families and showed positive outcomes regarding feasibility and acceptability [17]. The aim of the current case study is to highlight the coping and psychosocial changes in one family recruited after the initial pilot study. We will conclude with recommendations to improve policy initiatives for the public health and well-being of other Black American families regarding racial discrimination.

## 2. Materials and Methods

### 2.1. EMBRace Procedure

EMBRace is a RS intervention that seeks to: (1) *engage* Black American youth and their caregivers in conversations on race; (2) *manage* racial stress and trauma resulting from discriminatory experiences; and (3) promote parent–child *bonding* through the RS process. The intervention aims to help families feel more efficacious in discussing issues of race, thus increasing competency and reducing stress responses. The intervention was developed by an interdisciplinary team of psychology, social work, and education researchers and clinicians after a review of the RS literature. Team members worked collaboratively to outline session prompts and activities based on relevant literature and measures with several rounds of edits to the manual after the initial pilot and again after two subsequent cycles for clarity and more consistent practice across clinicians. Given the manualized nature of the program, each family receives the same prompts and activities, though the order in any given session may be rearranged based on pressing needs presented by participants. Clinicians received six hours of training on the manual and cultural competency and participated in weekly group supervision. The composition of clinicians ranged in race (e.g., White American, Asian American, Black American, etc.), sex (e.g., female and male), and years (e.g., 0–8) and proficiency (e.g., master’s-level trainees to licensed clinical psychologists) of training. Community members and former study participants also provided input regarding session content and flow and general recruitment strategies through focus groups. Additional information regarding the intervention can be found in a feasibility and acceptability study [17] and a forthcoming development paper [18].

EMBRace consists of five 90-min sessions that one youth and their caregiver(s) complete with an individual and conjoint therapist. The first and seventh meetings are pretest and posttest assessments, while the five weekly sessions focus on content of the intervention; thus, each family requires seven weeks to complete the intervention. The 120-min pre–posttest assessments utilize research assistants in the administration of quantitative, qualitative, and observational assessments to family members independently and conjointly when needed. EMBRace does not use a group format at any point in the intervention. Please see Table 1 for an overview of the EMBRace intervention sessions. 

### 2.2. Participants

Inclusion criteria required youth participants to be between the ages of 10 and 14 years old and at least one of their caregivers to self-identify as Black American. The selected age represents the prime socializing years for emerging adolescents [5]. EMBRace encourages weekly participation from the target youth and at least one of their caregivers, though sessions can be spaced apart if needs arise. The term “caregiver” is defined broadly and includes biological parents, grandparents, foster parents, adoptive parents, etc. Participants were recruited through various community-based channels including referrals by local child-serving institutional staff, churches, scholarship listservs, flyering at community events (i.e., block parties, festivals), social media, and word-of-mouth. In addition, community partnerships were established with nonprofit organizations that serve the target population. 

Prospective participants were contacted by either a community partner or team member, depending upon where contact was made, and offered an initial overview of the study and a flyer. Individuals were offered the opportunity to provide contact information to continue the enrollment process. Once a contact number was provided, an EMBRace team member called to schedule a baseline testing date. At pretest, participants were given a detailed explanation of the study procedures and supplied with an informed consent and assent. The study was approved by the institutional review board of the university where the study was conducted (IRB #823939). 

Twenty parent–child dyads enrolled in the pilot and ten completed the intervention. As discussed in the feasibility publication [17], our completion rate was within normal range for clinical interventions and was impacted by transportation, after-school, and other non-EMBRace-related concerns. The current study illustrates the experience of one family who completed the intervention and expressed great concern over recent racial experiences and the current racial climate. While their process and outcomes did not differ greatly from other families, there are clear examples from the intervention which adds to our understanding of stress and coping processes for racialized stress. Ben* (child) and B. E.* (parent) (* denotes participant’s self-selected pseudonyms) are a mother–son dyad that was recruited to the intervention through promotional materials through a university. B. E. is a 47-year-old woman who self-identifies as African American. She is a wife and mother to two African American boys, Ben and his older brother. She is actively involved in homeschooling her children, a decision that she indicated was intentional and an active form of agency in protecting her sons from racism at school, and recently obtained an advanced degree in education. Ben is her 14-year-old son who self-identifies as African American. He lives in a two-parent household with his mother (B. E.), father, and older brother. Ben is homeschooled and studies at the eighth grade level. The family lives in a suburban neighborhood outside of a major northeastern city in the United States with an annual income range of US$50,000–74,000. 

### 2.3. Study Design

Using a repeated measures QUAN + QUAL pre–post analysis design, we sought to assess change in RS, coping, and psychosocial well-being for the case family, with a particular focus on Ben as a vulnerable Black American male.

### 2.4. Data Collection

Data were collected at pretest and posttest through quantitative and qualitative assessments by undergraduate and graduate research assistants. As qualitative data were collected via responses to interview questions for pretest and posttest, responses to manualized clinical prompts were also collected as part of the sessions throughout EMBRace implementation. Participants utilized iPads to input their quantitative responses to surveys, while audio and video tapes recorded verbal and physical responses, respectively, to qualitative interviews and clinical sessions.

#### 2.4.1. Measures of Participant Change

Racial Encounter Mindfulness Self-Efficacy Scale (REMS; [19]). The four-item REMS measure seeks to capture in-the-moment coping abilities when families are actively discussing racial encounters. Participants were asked to rate their perceived ability to: (1) calculate the level of stress they feel; (2) locate that stress physiologically; (3) communicate the stress they are experiencing to either themselves or a trusted individual; and (4) facilitate relaxation by breathing deeply and exhaling in that moment. Participants rate their ability to perform each coping strategy on a scale from 1–5, with “1” indicating not very well to “5” indicating very well. REMS was used in the beginning of each session, which allowed for a week-to-week assessment of the participants’ perceived efficacy in managing stress. As this is not a published scale, psychometric properties are not available.

Perceived Stress Scale (PSS; [20]). The 10-item PSS is one of the most widely used measures for assessing the perception of stress. The PSS measures how individuals appraise life situations as stressful. The PSS surveys about experiences within the past month in which participants are asked how frequently they feel a given way. In a review of multiple studies assessing the internal validity, Cronbach’s alpha values were at least 0.70 [21]. Although age norms were not found for the PSS, the author indicates that the reading level is at a junior high ability and has encouraged it for use with child populations in a resource report [22].

Racism and Life Experience Scales-Brief (RaLES-B; [23]). The RaLES-B includes nine items which assess the frequency, intensity, and stressfulness of multiple dimensions of racism-related experiences, including the Perceived Influence of Race, Racism Experiences, Daily Life Experiences, Life Experiences and Stress, and Group Impact. Although the RaLES-B is traditionally used with adults, the author provided permission to utilize the scale with young adolescents. The Cronbach’s alpha value has been found to be very high (αs = 0.88–0.90) in prior research [24]. 

Mental Health Continuum Short Form (MHC-SF; [25]). The MHC-SF is made up of 14 items that measure the adult’s emotional, psychological, and social well-being. It is derived from the Mental Health Continuum Long Form (MHC-LF) that has 40 items. This scale measures the frequency of positive experiences and has shown excellent internal consistency (>0.80) in prior research [25].

Brief Problem Monitor (BPM; [26]). The BPM is a 19-item scale designed to assess youth Internalizing (INT), Attention Problems (ATT), Externalizing (EXT), and Total Problems (TOT). Scales comprise items from the Child Behavior Checklist for Ages 6–18 (CBCL/6–18), Teacher’s Report Form (TRF), and Youth Self-Report (YSR). Statements such as “acts too young/old for his age” are accompanied by a scale of 0 (being not true), 1 (somewhat true), and 2 (very true). The BPM is specifically made for multiple informants (e.g., parent and child) and has shown excellent reliability (α = 0.91) and consistency with the CBCL (*r* = 0.95) in prior research [27]. 

#### 2.4.2. Qualitative Assessments

Qualitative assessments were administered in pretest and posttest interviews. The qualitative assessment was administered to parent and child separately. The questions mirror parts of the quantitative measures, including prompts on the parent–child relationship, existing RS practices, experiences with racial discrimination, program satisfaction, and clinician preferences. Questions about program satisfaction were only included in posttest interviews after the participants had completed the intervention. Responses were used to assess program feasibility and satisfaction. Items in this study were based on both the assessment and clinical interviews throughout the intervention.

### 2.5. Data Analysis

Survey items were entered into Qualtrics (The Rocks NSW 2000, Australia) and downloaded into SPSS Version 21 (Armonk, NY, USA) [28]. As the case study methodology precludes inferential analysis, only the individual’s scores are depicted numerically or graphically. Interviews were transcribed by Trint Software and research assistants and coded by the first and second authors with NVivo 11 (QSR International, Melbourne, Victoria, Australia) [29]. As coding for unique transcripts was divided amongst the first and second authors, inter-rater reliability was not established. With an explicit focus on coping with racial encounters and the resulting well-being of a vulnerable youth, coding was developed for the corpus with the nodes “Racial Encounter Coping” and “Psychosocial Well-Being”, with subnodes displayed in Table 2. 

## 3. Results

### 3.1. Quantitative Findings

B. E. and Ben’s pre- and post-intervention scores can be found in Table 3. 

**Figure 1:**
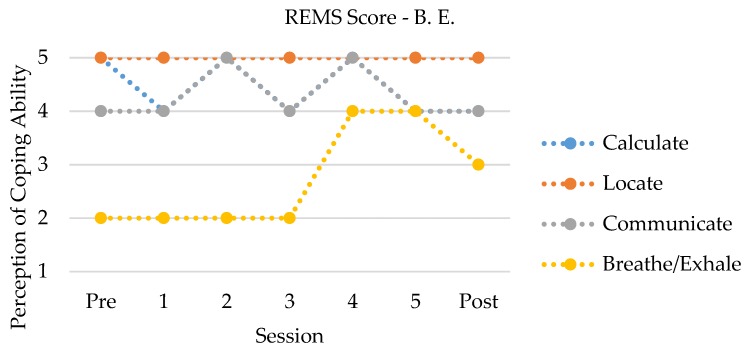
Parent responses to coping strategies through the duration of EMBRace.

For B. E., her self-report of both her general (PSS) and racism-related (RaLES) stress showed small decrements from pre- to post-intervention. B. E. endorsed a slight decrease in her overall racial coping self-efficacy (REMS) at posttest as compared to her ratings before the intervention. However, a more complex picture is provided by looking at the pattern of scores between sessions (see Figure 1). 

Specifically, B. E. showed the most improvement of her confidence in relaxing (breathe/exhale), while her perception of her ability to calculate and communicate seemed to vacillate between the two highest scores, and her ability to locate remained high throughout the intervention. With regard to B. E.’s own psychosocial functioning (MHC-SF), she showed increases in her psychological well-being. Lastly, looking at B. E.’s report of Ben’s psychosocial functioning (BPM), although already reported in the low range initially, B. E. indicated a modest decrease in report of attention and internalizing problem behaviors at posttest. 

**Figure 2:**
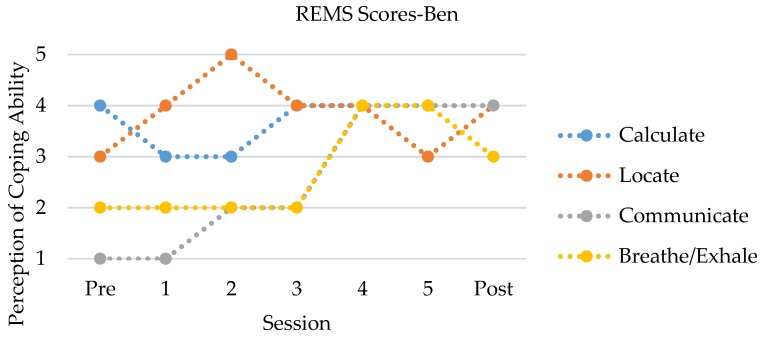
Child responses to coping strategies through the duration of EMBRace.

In contrast to his mother, Ben endorsed increases in both general and racism-related stress at posttest. Moreover, with regard to coping, Ben reported a marked increase in racial coping self-efficacy, from a score at pretest that indicated between “not well” and “a little bit”, to one at posttest that was nearly “pretty well”. Looking at these scores across sections (Figure 2), Ben showed the most improvement in his perception of his ability to relax and, importantly, to communicate. 

Notably, his perception of his ability to calculate showed an initial decrease across the first two sessions, before returning to and maintaining the pretest level. Lastly, Ben showed a negligible increase in his report of problem behaviors, indicating slight increases in attention, internalizing, and externalizing problems. 

### 3.2. Qualitative Findings

Ben and B. E. initially indicated some level of racial stress upon enrollment and explicated upon those concerns throughout the intervention. The following content was extracted from sessions to indicate the change in coping processes and their associated psychosocial correlates.

#### 3.2.1. Pretest

B. E. indicated a great deal of internalizing problems regarding both herself and Ben in the racialized society, particularly with regard to the current political administration. In response to what current concerns B. E. has for her child, she responded by noting:

Trump. As (Ben is) an African American male who will turn 18 in (Trump’s) administration, who I feel has a lot of potential and gifts. I am better today than I was in November and December (of 2016) when I had a lot of anxiety about our place in the world and just everything. But I’m not like I was—having anxiety—but I don’t know if I’m having that now, I’m just very sad and disappointed, and trying very hard in my daily journal to not write up a Trump contingency plan that involves me looking for jobs all over the world.

B. E. expressed her concern for her son but also quickly indicated how the internalization of her emotions manifested through her own anxiety. Her *racial knowledge* was evident in the discussion of well-being in relation to the way in which she anticipated her son to be perceived. 

Ben similarly described an incident when his knowledge of a racial encounter impacted his sense of self. With regard to what he would like his parents to do more of, he shared:

Ben: ...we don’t like need to like have race talks every single day, but like I think like once or twice a week is like, fine, like when something actually happens like even when I was coming out of my orchestra practice, there was—I was standing waiting for my mom’s car to pull up, so like next to (a prominent wealthy location), and there was...a family that was walking past and she was like ‘walk faster I don’t wanna get robbed’ and like I was like the only person standing there and she said it like really loud so I heard it. So it was just like, things like that, that I would actually talk about more.

Clinician: How did that make you feel?

Ben: Umm…not good, at all, like why would, if I’m holding an instrument in my hand and my hands are in my pockets why would you think I was concerned about anything that you were doing?

Clinician: Mmhmm. And did you speak to your parents, mom about this?

Ben: I told her and she was like ‘oh like, that’s sad’, and then we talked about it for a little bit but *shrugs*.

Ben also showed a desire to discuss this instance more fully, but, at the time did not receive the support he sought from his mother. Ben was able to utilize this encounter in subsequent dialogue with his mother throughout the intervention. 

#### 3.2.2. Session 1—“Say it Loud”

In the first session devoted to helping clients become more *knowledgeable* of racial encounters and their cultural heritage, B. E. described an instance in which she and her sister had recently discussed the possibility of an ancestral family member being killed by the member of the Ku Klux Klan:

My sister and I had this conversation within the last couple of weeks. I woke up one morning and wrote this freeform poem and shared it with her. And shared it with (my sons) and some of the stuff she didn’t even know. Because my mom would never really talk about it. So I feel better about it today. But I think it’s something that definitely isn’t over.

B. E.’s position—of being both aware of the situation (*self-other awareness*) and able to find catharsis from writing about it (*engagement*)—showed advanced elements of coping with racial encounters, and yet she still concluded with concern over what is to come. She also indicated a parallel to the sentiment raised by Ben in the prior session—that racial matters are only marginally addressed within the family, though the fear and resulting psychosocial well-being are impacted for years, if not generations. 

Ben similarly showed advanced coping processes by describing protesting as an active way of addressing discrimination (*engagement*) and indicated an *awareness of himself and others* after listening to the song “Say it Loud” by James Brown:

Clinician: What do you think it means to feel like you should “say it loud, you’re Black and you’re proud”?

Ben: Stand up for your own. Don’t be ashamed of your race. Because every race has issues and problems that they have to go through. I think every race—including the majority sometimes—can be discriminated against. But I feel like if you say it loud like it used to show that you support your race and what they’re going through.

Ben was able to achieve psychosocial wellness by taking a holistic perspective and keeping in mind what other people of varying races experience. 

#### 3.2.3. Session 2—“We Gon’ Be Alright”

In the second session, the family was expected to appraise their own or others’ emotions during a racial encounter, particularly as it related to being prepared for bias. B. E. described an encounter in college in which racial epithets were lodged at her when she sought advanced leadership positions and, when she attempted to file a complaint, was asked “what did (she) expect?”. She noted that she desired her peers and the administration to say, “‘Okay, you’re justified in your feelings or this is horrible’. (Instead) it was ‘get over, people are horrible’. You know I was just trying to don’t be sensitive about it—they’re horrible and just ignore it.” In this *appraisal* of her reaction in relation to others, B. E. indicated that there were inconsistencies between what she wanted to hear and what she felt and, thus, did not feel supported. 

In a second more recent encounter involving Ben’s friendships, B. E. described her son as “shocked” that peers would talk to him about taboo racial matters and desired for Ben to “have the skills to actually deal with it because that was just bad cuz I don’t even know I have that fully.” She further described her emotional ambivalence by appraising the way she felt when Ben’s advancement through school was challenged:

B. E.: The emotions of that them saying you know you don’t know what you’re doing with your kid and he’s not smart as you think he is. And all of that kind of stuff that. I feel like that was gaslighting in a lot of ways.

Clinician: I salute you for standing on top of that.

B. E.: I don’t know that I have…

B. E. concluded by indicating that she pulled Ben out of school and subsequently homeschooled him, but was not sure if that was actually engaging with the problem as much as it helped to alleviate the problem in the short-term. 

Ben described the desire to *engage* in a group discussion with other Black American students when racial matters came up, particularly so he could get a sense of their emotions in light of the “masks” he described people wearing and “walls” he described people hiding behind on social media sites (e.g., YouTube). He recalled one instance when a Black American former classmate was kicked out of school for an incorrect suspicion of theft. Ben noted that, in accordance with his mother’s sentiment, he was pulled out of school to protect him from the bias that his peers were encountering. While he said he could not be sure what his peer was expelled for, he felt as if it was likely due to race, thus *appraising* a situation as racial and his resulting emotions and schooling placement in relation to the encounter.

#### 3.2.4. Session 3—“I Got Enemies, Got a Lot of Enemies”

Session three, which focused on the active reframing of overgeneralizing beliefs about other races or groups, or promotion of mistrust, appeared to be useful for B. E. She spoke of an encounter from her early adulthood in which she was denied access to a leadership opportunity by her employer that still upset her decades later. B. E. described the process of *reappraising* the situation by offering this reframe of the scenario:

B. E.: So retelling the story: when I was there and you know having a conversation about being offered this opportunity. And. And being told that’s not for people like you. I probably would have asked for clarification, which I didn’t. I would probably have come in with alternatives—in ways that you know ‘I’m willing to do X in this or whatever with the kids or whatever around the house but this is something that I’m going to go to.’ And that’s not something that I’m asking your permission to go to. And you know just make it not so much negotiation not so much I am asking your permission to live my life, but this is what’s going to happen kind of switched around little things. I believe that I probably did a lot of asking when I probably didn’t need to.

Clinician: And you think that would’ve changed your level (of racial stress)?

B. E.: I think that it would have because it would be more empowering as opposed to me coming in because that was part of the agreement. If you need off, I need off you know that was the deal.

In this way, B. E. was able to more fully assert herself through her *reappraisal* of the situation. She reassessed her emotions and provided the way that she would more fully *engage* with the person who denied her access to the opportunity.

On the other hand, Ben expressed a relatively moderate view of the ways he had encountered the concept of promotion of mistrust. While he acknowledged that his family members or those in media may see entire groups of people in a certain light, he has had experiences with people who have counteracted that narrative. He provided a balanced approach when role-playing with the clinician the way in which some people may need to change their perspectives to more fully understand the circumstances of others. With regard to seeing how members within race can be viewed more positively, he noted with respect to his role-played grandfather:

Put yourself into their shoes...like when they grew up without any money in that household. Like all the money that they had, it was it was going to like...it was either being like coughed up in taxes or bills or something like that. So they didn’t actually have enough resources like in their school system...like it didn’t have like microscopes like that. So. So we didn’t actually have enough resources to be able to provide a good education.

In many ways, Ben’s advanced approach in this domain helped his mother in subsequent sessions to better understand just how negatively impacted he was by some of her less-balanced messaging.

#### 3.2.5. Session 4—“Does it Matter if You’re Black or White?”

In session four, the family was expected to continue using skills of *racial encounter knowledge*, *appraisal*, and *reappraisal* to discuss racial messaging that centers on egalitarian or colorblind ideologies. For B. E., Ben’s experience with prejudice allowed her to recognize both that her personal racial comebacks for dealing with being stereotyped as a “robber” may not have resonated for him *and* that it was important to be able to still engage with him to find a solution/racial comeback that would feel healthy:

And so I realized through that conversation that my normal response is you know some people are horrible. And I asked (Ben) if that made you feel better when I say screw em. And he said ‘it does not make me feel better’. Me just saying, you know ‘screw em—you know, they’re horrible. Don’t let them get to you. You know or something you know it doesn’t matter. You’ll see one day you’ll be their boss or something like that.’ So (Ben) said like that doesn’t help. So we kind of tried to talk through what would help and he was like ‘you know I said you know maybe we could sue them and you get a check’.

B. E. also seemed to identify the difficulty in choosing to engage with others who may be ignorant about their prejudices, similarly considering the toll that such discussions might take on her and her son. It seemed in this session that B. E. was better able to accurately appraise her thoughts and feelings. Through reflecting on and *reappraising* these experiences through *stress management* techniques, she expressed being able to better understand the importance of helping Ben identify healthy ways to cope with his own racial encounters, despite still expressing some doubts about her self-efficacy in this domain.

Notably, Ben began the session by describing increased benefit in utilizing racial coping strategies from REMS. “Well I think it’s been helpful for me. Like with my mom to be able to go over like important subjects like these because I don’t think we would have went over them as much.” Moreover, Ben seemed to feel empowered that he was actually able to share and work through an issue that had happened to him personally. Regarding the aforementioned “hurry up—I don’t wanna get robbed” incident he noted: “I really like this past week’s (session). Where we talked about, because I actually got to express one of my issues. Issues that happened to me. With my mom, we were able to talk about that.”

Unlike some prior sessions wherein Ben seemed slow to express himself, he openly shared a great deal of opinion—and *knowledge* of racial politics—pertaining to racial colorblindness and egalitarian perspectives. Importantly, in sharing his opinions, Ben not only showed an increased *awareness* of racial issues, but was also able to consider how he might feel afraid of saying the wrong thing in certain racial moments:

Clinician: And when you feel like you’re afraid of saying something wrong—how does that affect what you actually say?

Ben: Um. I think I start to start to like say I started talking a little slower. And I try to craft what I’m saying instead of just blurting it out...like right now I’m just saying what I’m thinking. If an issue were to happen or a situation like that I would start to slow down and be like okay so how do I say this without making them upset but I still want to get my point across.

He also comfortably *appraised* his stress upon considering having to debate the importance of race:

Clinician: What kind of stress do you think that brings a lot of people of color who have to (debate out the blue)?

Ben: Like that would like instantly jump me to a five (regarding stress levels from 0–10). Like it could be like oh wait. Why are we debating right now? Like I was not prepared for this...like instantly bring me up. Yeah.

As such, Ben was able to express himself through practicing his strategies in a safe environment. 

#### 3.2.6. Session 5—“Ain’t No Stopping us Now”

Session five served as the final active treatment session, providing both Ben and B. E. an opportunity to review the skills they had learned throughout the intervention. As all families are asked to do for this session, Ben and B. E. charted their stress over the course of the EMBRace intervention. For B. E., reflecting on her time in the program led her again to acknowledge the ways in which her “constant elevated anxiety” about the climate of the United States of America was impacting her parenting. However, she indicated that she had recently begun to enact self-care: disengaging from social and news media. During her graphing activity, B. E. again showed an *awareness* of the ways in which reflecting on her previous experiences in the sessions were impacting her stress, as well as her parenting decisions. In response to the inquiry, “What do you notice about the difference in scores between weeks 1 and 5?”, B. E. indicated:

B. E.: I can say it’s interesting: when I come in I’m usually at a 2. Last week when I left I was at a 2, but weeks before that, like week 3, I came in at a 2 and left at a 7...I think I’ve tried to make some deliberate (changes), I mean, I wouldn’t be homeschooling. Like all those things ended up being, ok, what can I do to kind of change that around.

With regard to her interactions with Ben, B. E. indicated that she noticed an *increased awareness and expression of feelings* and *engagement* from him, while also recognizing that she feels better knowing that certain lessons she has provided have been internalized:

And I like that—I like that correction ‘cause it’s like we don’t *know* the answer, but it’s probably a combination, that he’s definitely taking things, and I think, you know we’ll have more all together to reflect on what we, what (Clinician) and I have seen so, I definitely think in some ways that he’s definitely been engaged with this stuff and also there’s been some nice moments like last week where we were able to see like wow, he’s still recalling stuff from even before EMBRace, that predates EMBRace, about this idea about the myths of needing like, you know, like, you just pull yourself up by the bootstraps ‘cause Mom you told me like if you’re part of the problem you’re not part of the solution. So I think it’s both your being more aware and him being engaged, but also communicating your feelings.

While feeling a similar lack of confidence in racial comeback lines as her son, B. E. demonstrated that in the future she would be more willing to problem-solve around these topics with him.

Additionally, Ben provided an illustrative example of the ways in which practicing his skills had allowed him to *appraise*, *reappraise*, and *cope* with racially stressful matters:

Ben: …Like every week that I’ve been actively monitoring like my stress levels and stuff like that. I’ve started to come up with different strategies and that’s been helpful to like calm myself down or just like not talk to someone just like that.

Ben also indicated that engaging in these topics was not only useful in the short-term, but would also serve him well in the future: “I feel like it’s been good to actually be able to get these talks in somehow. Because I will be going to college pretty soon. And I will be on my own for a couple of years.”

Ben was adept at not only recalling his stress levels across the five weeks, but also in remembering critical incidents that contextualized his stress, such as a one-sided debate with his mother or feeling that his racial comebacks were inadequate. Consistent with the objectives of this session, Ben was also able to demonstrate an ability to *recast* and *resolve* the incident involving the couple who feared getting robbed, landing on a solution of looking back at the couple that, while different from his mother’s solution, felt right for him. Lastly, Ben was able to admit that while he still had some discomfort being expressive, continuing to practice had made it easier for him to share his feelings, and predicted that he would feel more able to express himself more quickly, both with regard to engaging with his mother, and in identifying racial comebacks.

#### 3.2.7. Posttest

During the posttest, although B. E. still indicated that she was concerned about the impact that the sociopolitical climate would have on her family, she noted that *engaging* in the conversations with Ben had allayed some of her anxiety:

Since the program has started, the anxiety level is lower. I think that that has something to do with the fact that it’s not so close to January 20th (2017) and the inauguration than it was. But also through our conversations with (Ben), ‘cause I said that, the first week or two, I’m not trying to feel like I’m constantly kind of walking around saying, that we’re not having conversations about race, that we’re having conversations about schoolwork. But I just felt I was doing a lot of nagging. And nobody was listening to me and everybody was rolling their eyes, and ‘there she goes again’. But through the conversations with (Ben) here, I think my anxiety—and *I recognize it as anxiety, not so much parenting* (emphasis added)—or like “I need to get these things in,” ‘cause he’s not gonna be in my house forever—but I heard that he does listen to me, and I always wasn’t sure about that, which is crazy to say, but I learned that he does listen and respect my opinion. And I wasn’t entirely confident that that was happening. So I think that—whenever I did, as I kind of went through my life, it doesn’t feel as latent or as pressurized. It is, I have this finite amount of time and it feels more conversational, you could say? Natural.

Finally, Ben shared that he and his family had shifted from talking about race in a secondary manner, “because of other things … like something that is happening in the Oval Office”, to *engaging* more explicitly, “actually having discussions on race”. He also endorsed a commitment to improving his rumination (“sometimes I just let it sit”), noting that he would like to get his thoughts out to his family sooner after incidents occur. Notably, the desire Ben showed at pretest to discuss racial incidents with his parents remained, but now seemed accompanied by a greater ability to navigate such conversations, with Ben indicating that he felt that his outlook on discrimination had been “strengthened”.

## 4. Discussion

Although a number of studies indicate that RS is associated with psychosocial well-being (e.g., [5], few interventions have been developed to incorporate this “natural” strategy into practice with Black American families. Thus, the purpose of this study was to examine whether a RS intervention (EMBRace) was useful at enhancing coping strategies for Black American families to reduce internalizing and externalizing problems in youth vulnerable to the effects of racial discrimination. Both the qualitative and quantitative results indicate that coping changes were present for Ben and B. E., which led to improved psychological wellness. Quantitatively, Ben showed an overall increased confidence in his ability to calculate, locate, communicate, and relax during racially stressful moments, while B. E. showed a slight decrease, from pre- to post-intervention. It appears that Ben felt increasingly efficacious in his ability to communicate and relax throughout the intervention. In addition, he exhibited a slight decrease in his ability to calculate during the first two sessions of the intervention but was able to indicate increased self-efficacy afterward. B. E. also showed an increased ability to relax with continued involvement in the intervention, with scores in the other domains that alternated between the two highest scores possible. Qualitatively, Ben and B. E. both described moving from less concrete to more active forms of coping, particularly through enhanced *racial encounter knowledge*, *stress management*, and *coping techniques*. Ben and B. E. described the ways in which the elements of EMBRace (e.g., engaging, managing, and bonding) were apparent within the sessions and helped them to discuss racial experiences more competently and confidently. 

The hypotheses posited for this study were that EMBRace would result in an improvement in RS competency, improved coping strategies, and reductions in psychological distress. Our findings support the hypotheses through qualitative and quantitative means. With regard to competency and coping, as stated previously, both Ben and B. E. showed an increased self-efficacy in their ability to relax during racially stressful moments, with Ben also showing marked improvements in his confidence in communicating during these encounters. Notably, the pattern that Ben reported with regard to his ability to calculate his level of stress has likewise been demonstrated by other participants [16]. Although no statistical inferences can be made regarding the changes in psychological distress, B. E. indicated a slight decrease in her report of Ben’s problem behavior, particularly attention and internalizing behaviors, while Ben indicated a slight *increase* in his own attention, internalizing, and externalizing behavior. Two points are worth noting here. First, previous research has typically shown only moderate associations between parent and child report of behavior problems [30]. Moreover, this study showed that parental symptoms are related to their report of their children’s symptoms. In this case study, B. E. showed improvements in her own psychological well-being, which may have also impacted how she saw Ben’s functioning. Second, Ben indicated a dramatically improved confidence in communicating his thoughts and feelings. Although the questions were in the context of race-related encounters, this improved “voice” may have also led to a great confidence in endorsing symptoms he may have been struggling through. Additionally, qualitative analysis reflected more explicit racial conversations that were bidirectional, the deployment of active coping strategies (e.g., “unplugging”, starting a dialogue), and both noticing and reducing psychological problems by both parent (e.g., anxiety) and child (e.g., fear). These findings are consistent with the RECAST model [3] and other suggestions for applied methods of coping improvements with racialized experiences [31].

Taken together, these findings contribute to advancing the literature on the mechanisms involved in RS practices. By applying RS processes through an active component, that is, an intervention, we can better determine a possible causal link between RS and outcomes. In particular, that coping strategies were enhanced throughout the intervention identifies a potential pathway by which outcomes are being changed [3,32]. The improvement of racially-specific coping strategies through RS is akin to the ways in which general coping strategies and subsequent youth well-being are enhanced through general coping socialization [33,34]. Thus, intentional and deliberate communication of content specific to the stressor may be an important consideration for Black American youth who face racial stress. Additionally, such findings lend themselves to the clinical significance of addressing culturally-related stressors with clients and as such is important in the intentional cultivation of cultural competence in clinicians. Moreover, it is critical to consider the ways in which policy initiatives can support the redressing of historical and current racial discrimination in the United States. Several levels of intervention have been discussed that impact the macro to micro systems impacting Black Americans [35]. In tandem with health-enhancing family-level programs like EMBRace, it is important to improve the educational, employment, and residential opportunities available to Black Americans in direct response to generations of oppression for an exponential effect [35,36]. Federal funding should continue to support interventions which can reduce the impact of racial discrimination in various sectors for Black American health and wellbeing.

Although this case study has the potential to inform treatment practices for Black American youth exposed to stress from discrimination, there are some limitations worth noting. Ben and B. E. were recruited via word-of-mouth, thus their desire and willingness to participate was high. Furthermore, that results are from a case study, they cannot be analyzed inferentially nor systematically. As such, results from the other pilot participants will be important to analyze with regard to their similarities and differences to Ben and B. E. Finally, Ben and B. E. represent only two members of their nuclear family: results may have differed if the father or other child were involved in the study.

Those elements notwithstanding, this case study highlights the way in which theorized concepts can be actively improved upon, particularly in light of the current sociopolitical climate in the United States. While the Civil Rights Movement and legislature of the 1960s depended on calculated and coordinated efforts, the present-day activism of the Black Lives Matter movement is much more fast-paced and decentralized. As such, modules and practice guides may be especially relevant in moving from research to practice and practice back to research. In residential areas that experience heightened stress after a racially-charged encounter, the active and timely employment of clinical strategies, particularly by therapeutic agents (e.g., therapists, lay counselors, parents, etc.) is of the utmost importance [37]. Thus, elements of EMBRace which have been associated with improved coping and outcomes (e.g., practicing “comeback” lines, engaging in healthy debating, etc.) may be especially useful for active resistance to discrimination in the moment. 

Future research should seek to understand the impact of EMBRace with larger samples, particularly in relation to dyads with varying sex pairings (e.g., mother/son, father/son, mother/daughter, father/daughter, etc.). The literature is clear on the different strategies utilized by different caregivers (e.g., [5,38]) so examining these effects in an intervention would be useful for both practical and empirical purposes. Forthcoming research should also include the physiological response to both discriminatory experiences and coping strategies for a more cohesive biopsychosocial depiction of stress and coping in participants. To best understand these additional variables of consideration, a multi-method randomized controlled trial design to assess the findings from EMBRace relative to a control group is anticipated. 

## 5. Conclusions

In sum, this case study shows the continued promise of the EMBRace program as a family-level intervention for improving the psychological well-being of Black American youth through enhanced, culturally-relevant coping. Given the ongoing onslaughts that these vulnerable youth continue to encounter in society, it is our fervent desire that the skills taught in EMBRace will serve as metaphorical armor, preparing families to engage in and overcome the persistent challenging discriminatory encounters still faced by many Black American youth today. 

## Figures and Tables

**Table 1:** Overview of the EMBRace intervention: targeted skills, content, and example activities.

Session	Engaging in RS Content	Managing Stress	Bonding and Effective Delivery	Example Activities
Pre-test	Baseline Assessment			
Session 1 “Say it Loud”	Cultural Socialization	Racial Encounter Knowledge	Increase parental warmth and interconnectedness through shared racial/cultural experiences and heritage Deliver parenting and familial behaviors with affection	Create a family tree that includes people, places, and traditions important to the family
Session 2 “We Gon’ Be Alright”	Preparation for Bias	Racial Encounter Knowledge	Deliver parenting and familial behaviors with protection	Complete a mock debate examining the importance of preparing youth for discrimination
Session 3 “I Got Enemies, Got a Lot of Enemies”	Promotion of Distrust	Racial Encounter Stress Management	Deliver parenting and familial behaviors with correction	Practice racial storytelling and narrative sharing on a racial encounter from the past
Session 4 “Does it Matter if You’re Black or White?”	Egalitarianism	Racial Encounter Stress Management and Coping	Deliver parenting and familial behaviors with connection	Role-play responses to egalitarian messages in various settings
Session 5 “Ain’t No Stopping Us Now”	Applied Skills	Racial Encounter Stress Management and Coping	Deliver skillful parenting practices with affection, protection, correction, and connection	Integrate four tenets of RS for increased competency
Post-test	Post-Intervention Assessment			

Note: EMBRace = Engaging, Managing, and Bonding through Race; RS = racial socialization.

**Table 2:** Nodes and subnodes of qualitative coding.

Coding Nodes and Subnodes	Definitions
*Racial encounter coping*	The cognitive and behavioral efforts made to master, tolerate, or reduce external and internal demands and conflicts among them.
*Racial encounter knowledge*	An individual knowing that a racial encounter is occurring.
Self and other awareness	Observing oneself within the racialized moment and becoming aware of others.
*Racial encounter stress management*	The management of stress in the midst of a racial encounter.
Stress appraisal	The appraisal of one’s level of stress, which involves both the recognition that the encounter is racial and that it creates in one’s self and others cognitive, emotional, and physiological reactions during and after the encounter.
Stress reappraisal	The reappraisal of the encounter after engaging in stress-reduction techniques, particularly for reframing a situation to see it in a positive light.
*Racial encounter coping*	The cognitive and behavioral efforts made to master, tolerate, or reduce external and internal demands and conflicts among them.
Engagement	One’s ability to engage—rather than avoid—to best cope with racial encounters.
Resolution	Individuals making healthy decisions that are neither an under- or over-reaction during and after racial encounters.
*Psychosocial Well-Being*	The interaction of psychological and social factors related to functioning and wellness (e.g., physical and mental).
Externalizing problems	Undesirable behaviors directed outwardly, including conduct problems, aggression, disobedience, etc.
Internalizing problems	Undesirable behaviors that are directly inwardly, including anxiety, depression, fear, psychosomatization, etc.
Wellness	Desirable functioning that can be inward and outward elements of mental and physical health, including autonomy, acceptance, growth, accomplishment, etc.

**Table 3:** Parent and child pre- and post-intervention scores.

		B. E. (Parent)		Ben (Child)	
Construct	Measure	Pretest	Posttest	Pretest	Posttest
Experiences with RD ^1^	RaLES ^2^	3.67	3.11	3.44	3.56
General stress	PSS ^3^	3.40	3.10	2.8	3.2
Psychosocial—Parent	MHC-SF ^4^	55	58		
	MHC-SF EWB ^4a^	15	15		
	MHC-SF SWB ^4b^	15	16		
	MHC-SF PWB ^4c^	25	27		
Psychosocial—Youth	BPM TOT ^5^	1.11	1.0	1.26	1.63
	BPM ATT ^5a^	1.17	1.0	1.0	1.67
	BPM INT ^5b^	1.17	1.0	1.5	1.67
	BPM EXT ^5c^	1.0	1.0	1.29	1.57
Perception of coping	REMS ^6^	4.5	4.25	2.5	3.75

^1^ RD = racial discrimination; ^2^ RaLES = Racism and Life Experiences Scale; ^3^PSS =Perceived Stress Scale; ^4^ MHC-SF = Mental Health Continuum-Short Form; ^4a^ EWB = Emotional Wellbeing; ^4b^ SWB = Social Wellbeing; ^4c^ PWB = Psychological Wellbeing; ^5^ BPM = Brief Problem Monitor, TOT = TOTAL; ^5a^ ATT = Attention Problems; ^5b^ INT = Internalizing Problems; ^5c^ EXT = Externalizing Problems; ^6^ REMS (Racial Encounter Mindfulness Self-Efficacy Scale.
